# Practical Notes on Diagnosis and Treatment

**Published:** 1910-10-15

**Authors:** 


### Practical Notes on Diagnosis and Treatment.

### Intestinal Obstruction.

If flatus and fæces both fail to pass during a period of twenty-four hours, in spite of repeated enemas, the diagnosis of intestinal obstruction is established and should be acted upon immediately. *Mr. P. Daniel.*

### Incomplete Abortion.

Although not infrequently successful, ergot is really contra-indicated in cases of incomplete abortion. In the cases in which it fails to secure expulsion of the retained fragments—and these cases are the majority—it is responsible for much loss of time and consequent increased risk of septic infection. The proper treatment for these cases is exploration of the uterus under an anæsthetic.—*Dr. Victor Bonney.*

### Beverages for the Gouty.

Rough cider, that is the completely fermented apple juice, agrees well with most gouty subjects. It contains but a small percentage of alcohol, is free from sugar, and its acidity is chiefly due to malic acid, which passes into the circulation in the form of alkaline malates, which in their turn are converted in the kidneys into alkaline carbonates, and excreted as such, thereby increasing the elimination of urates. The bottled or champagne cider should never be used, owing to its undoubted liability to set up gastro-intestinal fermentation. Dry perry is also a suitable drink for the gouty.—*Dr. A. P. Luff.*

### Laryngismus Stridulus.

Attacks of laryngeal spasm in infants and young children have on several occasions been found to be associated with elongated uvulæ.—*Dr. Abercrombie.*

### Thyroid Extract.

Amongst the conditions for which thyroid extract has proved useful are threatened abortion and subinvolution of the uterus. It has also some value as a galactagogue, and its use has been followed by shrinkage of uterine fibroids.

### Phthisis.

On waking in the morning a tumblerful of milk should be taken, mixed with a little hot water, to which it is often useful to add a few grains of common salt and bicarbonate of soda, especially when an accumulation of mucus has to be got rid of. Sometimes, in advanced cases, the stimulus of a tablespoonful of brandy or whisky in addition is needed at this time.—*Dr. Burney Yeo.*

### Abrasion of the Cornea.

The conjunctival sac must be washed with warm boracic lotion, and atropine ointment (1 gr. to the ounce) inserted between the lids. The eye should be kept tied up until the surface has healed. If there is photophobia, a shade may be worn over the good eye, or dark glasses used, a pad of wool being placed between the glass over the bad eye sufficiently thick to keep the lid closed.

